# A phase II study of buparlisib in relapsed or refractory thymomas

**DOI:** 10.3389/fonc.2022.891383

**Published:** 2022-10-18

**Authors:** Mohammad I. Abu Zaid, Milan Radovich, Sandra Althouse, Hao Liu, Aaron J. Spittler, Jeffrey Solzak, Sunil Badve, Patrick J. Loehrer

**Affiliations:** ^1^ Department of Medicine, Indiana University Melvin and Bren Simon Comprehensive Cancer Center, Indianapolis, IN, United States; ^2^ Caris Life Science, Dallas, TX, United States; ^3^ Department of Biostatistics, Emory University, Atlanta, GA, United States

**Keywords:** buparlisib, PI3Kinase inhibitor, thymoma, thymic epithelial tumors, phosphoinositide-3-kinase/Akt (PI3K/Akt) pathway

## Abstract

**Purpose:**

To investigate the efficacy and safety of buparlisib, an oral pan-PI3K inhibitor, in relapsed or refractory thymomas.

**Methods:**

This was a single center, single arm, open label phase II trial of buparlisib in patients with recurrent thymoma who have progressed after at least one prior line of treatment. The primary endpoint was objective response rate (complete response [CR] + partial response [PR]). Secondary endpoints included toxicity; progression free survival (PFS); overall survival (OS); disease control rate (DCR), i.e., the percentage of patients who achieve either PR or CR or stable disease [SD] for at least 4 months.

**Results:**

Between 10/13/2014 and 1/18/2017, 14 patients with stage IV disease were enrolled. Median age was 58y (23–74). 71% were females and 71% white. All patients had WHO B2 (29%) or B3 (71%) thymoma. Patients received buparlisib for a median of 4.5m (2–33). At a median follow up of 16.6m (2.4–31.3), onr patients (7%) achieved a PR. DCR was 50%. Median PFS was 11.1m (95% CI 2.9 – 18.8). Median OS, updated as of March, 2021 was 22.5m (10.7–31.3). Most common grade 3-4 adverse events related to buparlisib were dyspnea (21%), rash (14%), elevated transaminases (14%), cough (7%), pneumonitis (7%), anxiety (7%), fatigue (7%) and hyperglycemia (7%). Reasons for treatment discontinuation included progression of disease (n= 5), rash (n=4), pulmonary toxicity (n=3), sinusitis (n=1), and disseminated toxoplasmosis plus autoimmune cholangitis (n=1). As of 3/2021, 8 patients have died, 7 due to disease progression and 1 due to central nervous system toxoplasmosis and autoimmune cholangitis.

**Conclusion:**

Buparlisib showed modest activity in patients with relapsed or refractory thymomas. Further investigation of PI3K pathway targeted therapy in thymoma is warranted. (clinicaltrials.gov ID: NCT02220855).

**Clinical trial registration:**

clinicaltrials.gov, identifier (NCT02220855)

## Introduction

Thymic malignancies are rare but represent the most common tumors in the anterior mediastinum (1). Thymic epithelial tumors (TET) originate from the epithelial cells within the thymus and are histologically classified into WHO types A, AB, B1, B2, B3 thymoma, and thymic carcinoma. Whereas types A, AB and B1 thymomas have a better prognosis than the other histologies, all of these tumors are felt to be malignant and thus capable of producing metastasis and death. Management is multidisciplinary in nature with complete surgical resection the primary means for long-term survival.

In the setting of advanced thymoma or thymic carcinoma, the chance of cure is remote, and systemic therapy is typically employed with or without radiation therapy. Evidence-based guidelines favor combination chemotherapy regimens in the first line, usually containing a platin agent and some combination of doxorubicin, cyclophosphamide, paclitaxel, or etoposide (2–4). Unfortunately, given the rarity of the disease, there have been few prospective trials of systemic therapy in the advanced or metastatic settings. Using platin-based combination chemotherapy, response rates in first line therapy for locally advanced or metastatic disease are good are over 50% as reported in multiple phase II studies but few produce durable remissions (5).

As such, the vast majority of patients with locally advanced or metastatic thymic malignancies ultimately fail initial therapy and therefore require second-line therapy. Few agents have been investigated for these patients with variable rates of response (10-20%) which are rarely durable. Examples of these agents include: octreotide ± prednisone, imatinib, pemetrexed, belinostat, gefitinib, everolimus and interleukin-2 (5). There is a clear need for better therapies, especially those that can target specific mutations (6).

Given the infrequency of these tumors, the use of established tumor cell lines has been invaluable to evaluate potential new targets for therapeutic trials. One of these targets is the phosphoinositide-3-kinase/Akt (PI3K/Akt) pathway. Prior published data from our group demonstrated that a subset of thymomas activate the PI3K pathway through upregulation of a large microRNA cluster on Chromosome 19 (7). We have also demonstrated that the majority of thymomas have an active pathway as evidenced by elevated phospho-AKT compared to normal tissues (7). Albertobello and colleagues demonstrated that a mutation in the gene, PIK3R2 (that encodes a regulatory subunit of PI3K) and other PI3K subunit genes were found in a newly established thymic carcinoma (MP57) cell line, where inhibition of PI3K with GDC-0941 resulted in anti-tumor activity (8). Another independent group also observed an activated PI3K pathway at the protein level in thymoma tissues (9). Further support of targeting the PI3K pathway comes from a Phase II trial in thymoma patients with the mTOR inhibitor, everolimus. This demonstrated a disease control rate in 30 of 32 (93.8%, 95% CI, 79.2% to 99.2%) including three (9.4%) PRs (10).

Supported by this preclinical data, we initiated a Phase II trial of the oral pan-PI3K inhibitor, buparlisib, in patients with advanced thymoma (clinicaltrials.gov ID: NCT02220855). Buparlisib has potent inhibitory activity of all PI3K isoforms: alpha, beta, gamma, and delta. The primary objective of this trial was to evaluate the objective response rate, with secondary objectives being progression free survival, duration of response, toxicity, and overall survival.

## Methods

### Trial design and patient selection

We initiated a single-arm, Phase II trial of the oral pan-PI3K inhibitor, buparlisib, in patients with relapsed or refractory thymomas. The primary endpoint was objective response rate (ORR), with secondary endpoints of progression free survival (PFS), overall survival (OS), disease control rate (DCR), and toxicity evaluation. An exploratory objective to evaluate molecular markers of response was also planned. Eligible patients must have had with histologically confirmed thymoma (WHO Type A, AB, B1, B2, B3) with least one prior line of platinum-based chemotherapy (unless refused or not tolerated). Patients with thymic carcinoma (WHO Type TC) were excluded. Patients must have measurable disease, adequate bone marrow function (ANC ‗1.5 x 10^9^/L, platelets ‗100,000 x 10^9^/L, Hb >9 g/dl), liver function tests (ALT and AST WNL or ¾3 x ULN if liver metastases present, bilirubin WNL or ¾1.5 x ULN if liver metastasis present) and serum creatinine ¾ 1.5 x ULN. Patients with concurrent severe and/or uncontrolled concomitant medical conditions (e.g. cardiac, diabetic, gastrointestinal or pulmonary dysfunction). Pulmonary function tests including measures of predicted lung volumes and DLco were performed only for those patients in whom pulmonary dysfunction was suspected. Patients who were on chronic oral corticosteroids or immunosuppressive agents were not eligible. Buparlisib (also known as BKM120) was administered orally at 100mg QD for two or more months until disease progression or unacceptable toxicity. The study was initially designed to treat patients until disease progression or unacceptable toxicity for a maximum of one year. The study was amended later to allow treatment beyond one year for those with continued benefit from treatment.

### Statistical design

The primary endpoint of the study was objective response rate (CR+PR) according to RECIST 1.1 criteria for buparlisib monotherapy in patients with advanced thymomas. The sample size was calculated based on the objective response rate. A two-stage Simon’s Minimax design was used to test the null hypothesis of 10% objective response rate (p_0 =_ 0.10) versus a clinically meaningful response rate of 30% (p_1 =_ 0.30) with a two-sided alpha = 0.10 and a power of 90%. The first stage of the study was planned to enroll 16 evaluable patients with thymoma. If ¾ 1 of the 16 patients demonstrated an objective response, then no further patients would be accrued. If two or more of the first 16 patients have a response, then accrual would continue until 25 evaluable patients would be treated. If there are 2 to 4 patients with a response in the total of 25 evaluable patients, then the objective response rate would be considered as uninterestingly low, while if there are 5 or more patients of the 25 who have a response, then the study therapy would be considered as clinically sufficient to warrant further study in a phase IIb or III trial. Under the null hypothesis (10% response rate), the probability of early termination in this cohort is estimated to be 52%. The sponsor prematurely terminated the study due to discontinuation of the investigational drug program. Prior to this, a total of 14 patients are included for efficacy and safety analyses.

### Data analysis

Demographic and disease characteristics were summarized using descriptive statistics. As the study was discontinued by an external reason that was completely independent of the observed data, the primary outcome of objective response rate was calculated with 95% confidence interval without adjustment to the two-stage nature of the study design. Survival curves were estimated using the Kaplan-Meier method, with 95% confidence interval calculated using Greenwood’s method. Progression Free Survival was defined as the time from study start until progression or death, with censoring at last follow-up. Overall survival was defined as the time from the study start to documented death, with censoring at last follow-up.

### Molecular analysis

#### Whole exome sequencing

This trial was able to capture 10 matched pairs of tumor and peripheral blood. The concentration and quality of gDNA was assessed using Agilent 4200 TapeStation. A DNA Integrity Number (DIN) of five or higher was required to pass quality control. One hundred nanograms of DNA per patient sample were used to prepare single-indexed cDNA libraries using SureSelectXTHS Human All Exon V6 (Agilent). The resulting libraries were assessed for their quantity and size distribution using Qubit 2.0 (Life Technologies) and Agilent 2100 Bioanalyzer. Libraries pooled at 200pM were utilized for clustering amplification using HiSeq 3000/4000 PE cluster kit and sequenced with 2x75bp paired-end configuration on the HiSeq 4000 (Illumina) using HiSeq 3000/4000 PE SBS kit. A Phred Q-score was used to measure the quality of sequencing with more than 90% of the reads reaching Q30 (99.9% base call accuracy).

The sequencing data was first assessed using FastQC (Babraham Bioinformatics, Cambridge, UK) for quality control. The sequenced libraries were mapped to the human genome (UCSC hg38) using BWA MEM aligner in an alternate contig aware (alt-aware) manner followed by post alt-processing step with BWA kit. The PCR duplicates were marked using PICARD Mark Duplicates. The coverage across target regions was assessed using PICARD Collect Alignment Summary Metrics and Collect Hs Metrics. Quality control of sequencing and mapping results was summarized using MultiQC. Base quality score recalibration (BQSR) was performed using GATK Base Recalibrator and Print Reads to generate analysis-ready BAM files. GATK Mutect2 was used to generate somatic variant VCF files and Ingenuity Variant Analysis (Qiagen) was used to visualize variants. For copy number analysis, variants were called using CODEX2 (11). Standard quality control was used comparing matched patient samples.

### RT-PCR

RNA from 14 patients was isolated using Allprep DNA/RNA FFPE kit (Qiagen #80234). cDNA conversion was then performed using High Capacity RNA-to-cDNA kit at 1ug per patient (Thermo Fisher Scientific). MicroRNA experiments were run on 7900HT Fast Real-Time PCR system and expression was observed using TaqMan Gene Expression Mastermix and miRNAs 519d, 517a, and RNU48 as the endogenous (Thermo Fisher Scientific Assay ID: 47897_mir, 479485_mir, and 001006 respectively). Analysis was achieved using Sequence Detection System version 2.4 and RQ manager (Thermo Fisher Scientific)

## Results

### Baseline characteristics

The patients characteristics are illustrated in **Table 1**. This trial enrolled a total of 14 patients with relapsed or refractory thymomas. The median age was 58 years (23 – 74) with 71% being females. All patients had stage IVa (pleural metastasis) or IVb (other metastatic sites) disease. The patients were heavily pretreated with a median of 3.5 prior regimens (range 0-8) and the majority had prior radiation therapy.

**Table 1:** Subjects characteristics at time of enrollment to the study.

Characteristic	Value (n=14)
**Age,** median in years (range)	57.8 (23.0-74.5)
**Gender,** n (%)	
Female	10 (71%)
Male	4 (29%)
**WHO histological subclassification,** n (%)	
B2	4 (29%)
B3	10 (71%)
**Masaoka Staging,** n (%)	
IVa	6 (43%)
IVb	8 (57%)
**Presence of paraneoplastic syndromes,** n (%)	
Yes (myasthenia gravis (MG)-2; pure red cell aplasia plus MG-1; hypogammaglobulinemia-1)	4 (29%)
No	10 (71%)
**ECOG performance status,** n (%)	
0	14 (100%)
**Prior therapies**	
History of prior surgery	12 (86%)
History of prior radiation therapy	8 (57%)
Number of prior chemotherapy regimens, median (range)*	3.5 (0-8)
Prior platinum-based chemotherapy*	13 (93%)

ECOG, Eastern Cooperative Oncology Group; WHO, World Health Organization.

*one patient refused initial standard cisplatin therapy.

### Adverse events

All reported toxicities related to buparlisib are listed in **Table 2**. The grade 3-4 adverse events were dyspnea (21%), rash (14%), elevated transaminases (14%), cough (7%), pneumonitis (7%), anxiety (7%), fatigue (7%) and hyperglycemia (7%). One patient developed documented pneumonitis which resolved with corticosteroids but was removed from study. No patient had irreversible pulmonary fibrosis.

**Table 2:** Treatment Related Adverse Events.

	Grade of toxicities								
AE Term	1	2	3	4	5	Total	% Total	Grade 3-5	% Grade 3-5
Fatigue	8	2	1	0	0	11	79	1	7
Anorexia	8	2	0	0	0	10	71	0	0
Pruritus	6	4	0	0	0	10	71	0	0
Rash acneiform	3	4	1	0	0	8	57	1	7
Rash maculo-papular	0	1	1	0	0	2	14	1	7
Anxiety	3	3	1	0	0	7	50	1	7
Cough	2	3	1	0	0	6	43	1	7
Nausea	6	0	0	0	0	6	43	0	0
Dyspnea	1	1	3	0	0	5	36	3	21
Pneumonitis	0	4	1	0	0	5	36	1	7
Tremor	5	0	0	0	0	5	36	0	0
Depression	2	1	0	0	0	3	21	0	0
Dysgeusia	2	1	0	0	0	3	21	0	0
Hyperglycemia	2	0	1	0	0	3	21	1	7
Insomnia	2	1	0	0	0	3	21	0	0
Musculoskeletal and connective tissue disorder	3	0	0	0	0	3	21	0	0
Alopecia	2	0	0	0	0	2	14	0	0
Diarrhea	1	1	0	0	0	2	14	0	0
Dizziness	1	1	0	0	0	2	14	0	0
Dyspepsia	1	1	0	0	0	2	14	0	0
Endocrine disorders - Other, specify	1	1	0	0	0	2	14	0	0
Mucositis oral	1	1	0	0	0	2	14	0	0
Skin and subcutaneous tissue disorders - Other, specify	2	0	0	0	0	2	14	0	0
Vomiting	2	0	0	0	0	2	14	0	0
Weight loss	1	1	0	0	0	2	14	0	0
Non-cardiac chest pain	1	1	0	0	0	2	14	0	0


	Grade of toxicities								
AE Term	1	2	3	4	5	Total	% Total	Grade 3-5	% Grade 3-5
Alanine aminotransferase increased	0	0	0	1	0	1	7	1	7
Aspartate aminotransferase increased	0	0	0	1	0	1	7	1	7
Confusion	1	0	0	0	0	1	7	0	0
Constipation	1	0	0	0	0	1	7	0	0
Fever	0	1	0	0	0	1	7	0	0
Gastrointestinal disorders - Other, specify	1	0	0	0	0	1	7	0	0
Headache	1	0	0	0	0	1	7	0	0
Hypokalemia	1	0	0	0	0	1	7	0	0
Infections and infestations - Other, specify	0	1	0	0	0	1	7	0	0
Pain in extremity	0	1	0	0	0	1	7	0	0
Platelet count decreased	1	0	0	0	0	1	7	0	0
Psychiatric disorders - Other, specify	1	0	0	0	0	1	7	0	0
Respiratory, thoracic, and mediastinal disorders - Other, specify	0	0	1	0	0	1	7	1	7
Sinusitis	0	1	0	0	0	1	7	0	0
Skin infection	0	1	0	0	0	1	7	0	0
Edema limbs	0	1	0	0	0	1	7	0	0
Bronchial infection	0	1	0	0	0	1	7	0	0

### Efficacy results

**Figure 1:**
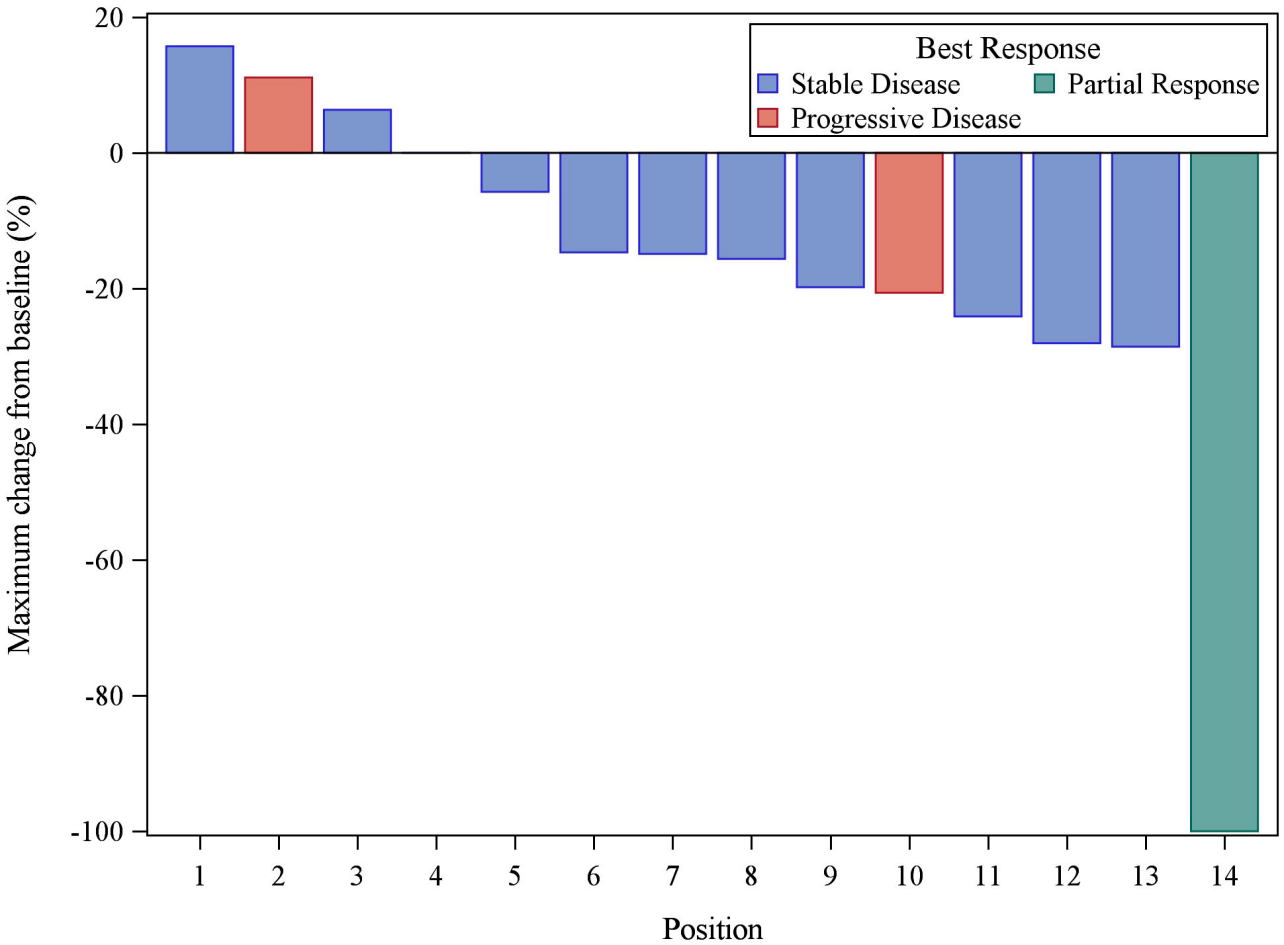
Waterfall plot of the 14 evaluable patients treated with buparlisib demonstrating best response of target lesions by RECIST criteria. One PR result was unconfirmed by follow-up scan (taken off study for toxicity).

**Figure 2:**
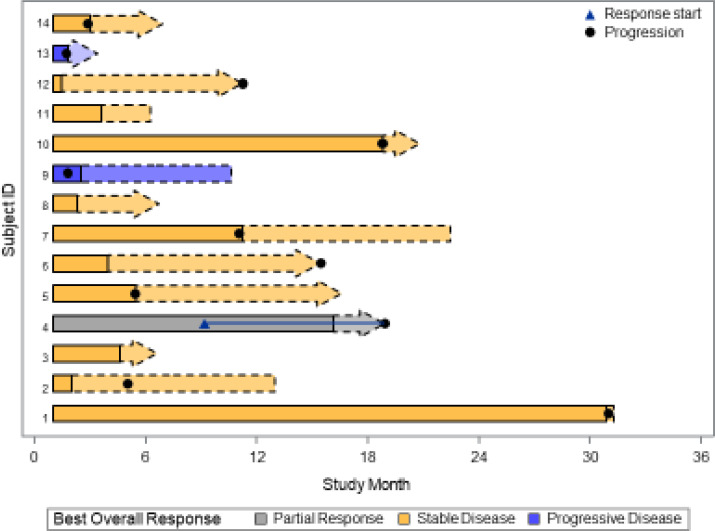
Swimmer plot of the 14 evaluable patients treated with buparlisib.

Patients received buparlisib for a median of 4.5 months (2 – 33). At a median follow up of 35.8 months (range 6.3 – 58.6 month), 1 out 14 patients (7.1% with 95% CI, 2%-42.8%) achieved a PR. DCR was 50%. The median progression-free survival (PFS) was 11.1 months (95% CI 2.9 – 18.8). As demonstrated in the waterfall plot shown in **Figure 1**, the majority of patients had some degree of regression with this agent. As of March, 2021, eight patients have died, seven due to disease progression and one due to central nervous system toxoplasmosis and autoimmune cholangitis. The median OS with the updated data was 40.0 months (95% CI, 28.3 – not reached) with the overall survival curve in **Figure 2**.

### Molecular analysis of the C19MC cluster and exome-sequencing

**Figure 3:**
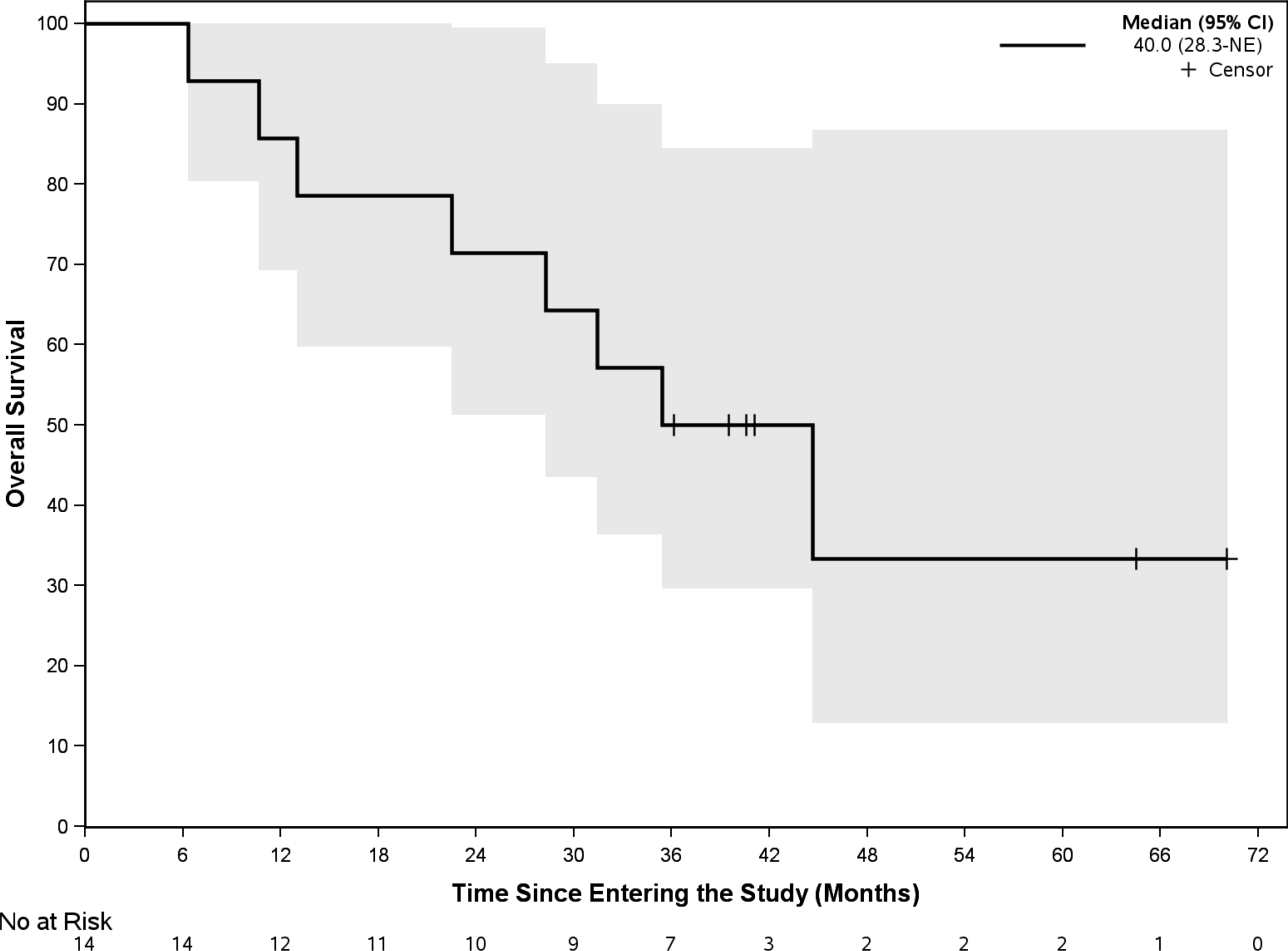
Kaplan-Meier survival curve of all evaluable patients.

In our previous studies, it was found that thymomas types A and A/B revealed a microRNA cluster on chr19q13.42 that was significantly overexpressed. With thymoma WHO subtypes being ambiguous, microRNAs 519d and 517a from this cluster were tested to evaluate activation status among the patients in this trial who have responded versus those that had progressive disease. In all patients that were tested, both mir519d and mir517a were observed to have little to no expression at the RNA level that would indicate that a patient may have been incorrectly subtyped pathologically (**Figure 3**).

#### Whole exome sequencing

Ten patients with matched tumor and blood samples were sequenced to observe any genomic variants that may explain the responses to buparlisib seen in two of the patients. Looking at SNVs we found no significant alterations that would explain sensitivity to PI3K inhibition. Further observation of copy number variation found a single amplification in PIK3CD in patient. This however did not meet the criteria of significance and could be described as an aneuploidy. Here we have found no significant genomic markers of response for buparlisib.

## Discussion

Therapeutic options for patients with recurrent TETs are limited. Whereas systemic cisplatin-based chemotherapy commonly produces remissions in previously untreated patients, most targeted agents have limited impact in previously treated disease. These include agents such as pemetrexed, sunitinib, cixutumumab and pembrolizumab (5, 12) with objective response rates between 6- 23%. In one of the largest prospective trials evaluating the mTOR inhibitor, everolimus, in recurrent TET (thymoma and thymic carcinoma), five partial and one complete responses were noted in 51 patients (CR + PR =12%) with 3 partial responses occurring in the 32 patients with thymoma (10). This current trial is the first proof of principle study that prospectively evaluated a PI3K inhibitor in thymoma. This trial clearly demonstrates clinical activity with buparsilib in patients with recurrent thymoma.

The rationale for evaluating this targeted therapeutic approach is based upon strong preclinical data. A unique microRNA cluster on chromosome 19 (C19MC) has been identified that is highly overexpressed in a significant subset of thymomas primarily comprised of the A and A/B subtype (7). We subsequently validated this observation in additional 35 thymomas by qPCR (7). This cluster is normally silent in adult tissues, with normal expression restricted to embryonic development. MicroRNAs within the C19MC cluster, miR-517 and miR-519d, have been previously demonstrated to inhibit key proteins in the PI3K/AKT pathway. Namely, PTEN, a negative regulator of AKT, and p21, a cell cycle arrest protein (13). In addition, gene expression analysis reveals over-expression of PIK3CA (aka PI3K p110), the canonical activator of AKT.To confirm, we performed a quantitative ELISA on a validation set of 35 thymomas for phospho-AKT (Ser 473), and demonstrate that C19MC positive thymomas have significantly higher levels of phospho-AKT compared to C19MC negative thymomas which in-turn has higher levels than adjacent normal tissue. Similarly, mutations of PI3K have also been observed in a new thymic carcinoma cell line, (MP57) (8).

**Figure 4:**
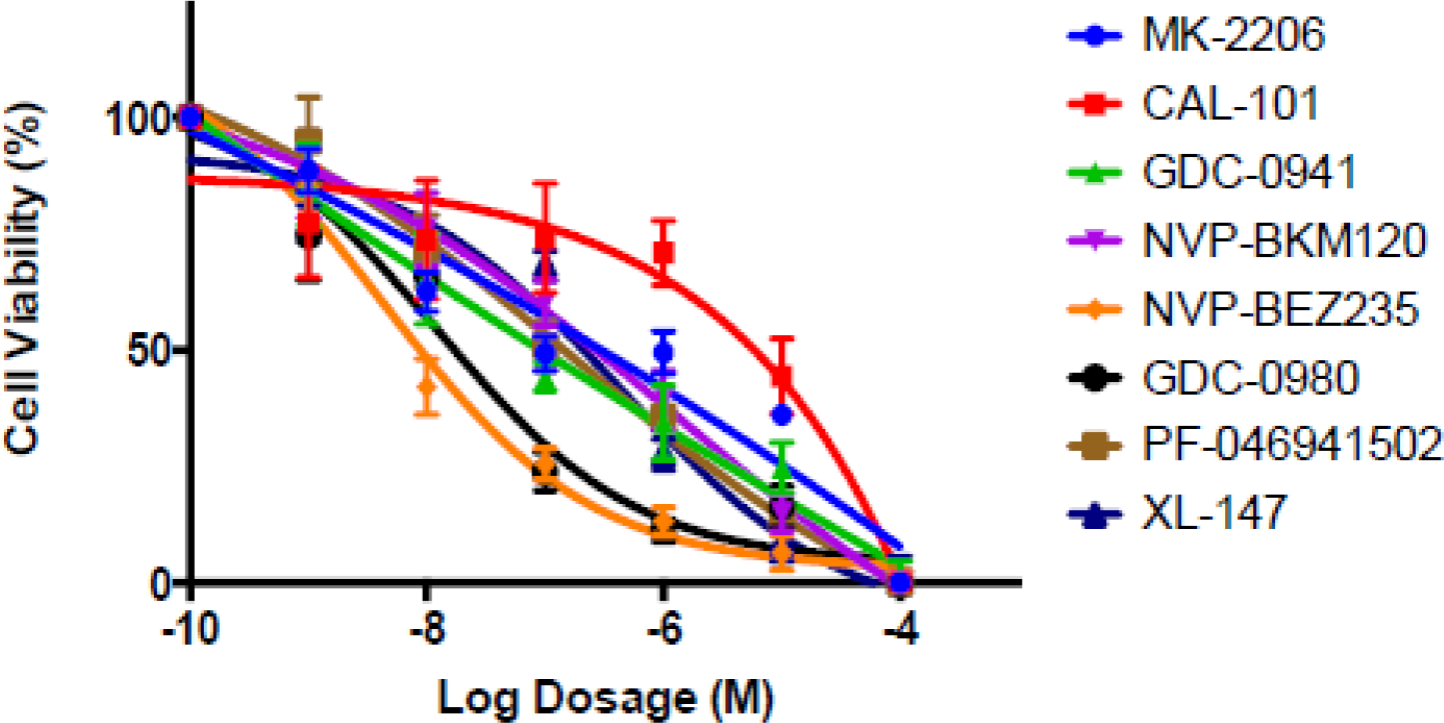
The thymoma cell line, IU-TAB-1 with increasing dosages of PI3K, AKT and dual PI3K/mTOR inhibitors. Cells were treated for 72 hours, and cell viability was assessed. The data demonstrates marked sensitivity of the cell lines to these inhibitors including Buparlisib (NVP BKM120).

These data demonstrate that TETs have the potential to have sensitivity to PI3K inhibition, in particular those that are positive for C19MC. We further tested a variety of PI3K, AKT, and mTOR inhibitors in our thymoma cell line (IU-TAB-1) and found significant activity for several of these agents (**Figure 4**). The serine-threonine kinase mammalian target of rapamycin (mTOR) is a key component of the PI3K/AKT/mTOR intracellular axis. Several investigators suggest that the PI3K/AKT network plays an important role in thymoma growth as mentioned above and may sensitize cells thymic epithelial tumors (TET) to mTOR inhibition (8, 14). This study confirmed that targeted blockage of the PI3k/AKT pathway has merit in advanced thymoma.

One of the limitations of this study was that all of the patients entered on the trial had WHO type B2 and B3 thymoma, whereas the molecular features suggest that the PI3K/AKT pathway may be especially important for WHO type A and AB thymoma. As the frequency of recurrent metastatic disease is more common in B2 and B3 thymoma, patients with type A, AB and B1 histology still develop recurrent metastatic disease but were not seen on this study. Another limitation was that the study was terminated early when the sponsoring company determined that they would no longer pursue this drug because of toxicities seen in other concurrent and previous trials. We did observe toxicity (notably dermatologic and pulmonary) that led to early discontinuation in several patients. Patients with thymoma have a high incidence of autoimmune disorders and altered immune systems making them more susceptible to opportunistic infections. In this series, one patient who was on study for 31 months developed autoimmune cholangitis and was started on corticosteroids, but died from disseminated CNS toxoplasmosis (documented by autopsy) many years after first contracting this disease. This patient was not neutropenic at the time of his diagnosis but died within one month of discontinuing study drug.

Early discontinuation was required in over half of the treated patients. Nonetheless, the results from this trial, demonstrate activity in thymoma among 14 patients treated on trial. All but five of the 14 eligible patients had some degree of regression while on therapy. Since initiation of this trial, three PIK3CA inhibitors have been approved by the FDA including, copanlisib (for refractory lymphoma); duvelisib (for refractory CLL and follicular B-cell lymphoma); and alpelisib (for refractory breast cancer) (15–17). Non-infectious pneumonitis and severe cutaneous reactions were noted to occur in ¾5-10% of treated patients. This trial provides evidence to support further evaluation of the targeting of PI3K/Akt pathway with novel and less toxic agents in patients with advanced thymoma.

## Data availability statement

The datasets used in this study are not publicly available due to privacy concerns. Requests to access the dataset can be directed to the corresponding author.

## Ethics statement

This study was reviewed and approved by Indiana University IRB. The patients/participants provided their written informed consent to participate in this study.

## Author contributions

MA – Protocol design, manuscript composition and review. MR - Protocol design, manuscript composition and review. SA – Data collection and manuscript review. HL - Statistical review and manuscript review. AS - Data collection, manuscript composition and review. JS - Laboratory analysis, manuscript composition and review. SB - Pathology review, manuscript composition and review. PL - Protocol design, analysis, manuscript composition and review. All authors contributed to the article and approved the submitted version.

## Funding

This work was supported by the National Cancer Institute Award CA082709. The authors also declare that this study received funding from Novartis in the form of the investigational drug. The funder was not involved in the study design, collection, analysis, interpretation of data, the writing of this article or the decision to submit it for publication.

## Conflict of interest

Authors MR and JS were employed by the company Caris Life Science.

The remaining authors declare that the research was conducted in the absence of any commercial or financial relationships that could be construed as a potential conflict of interest.​

## Publisher’s note

All claims expressed in this article are solely those of the authors and do not necessarily represent those of their affiliated organizations, or those of the publisher, the editors and the reviewers. Any product that may be evaluated in this article, or claim that may be made by its manufacturer, is not guaranteed or endorsed by the publisher.
